# Construction Land Expansion of Resource-Based Cities in China: Spatiotemporal Characteristics and Driving Factors

**DOI:** 10.3390/ijerph192316109

**Published:** 2022-12-01

**Authors:** Jiangsu Li, Weihua Li, Bo Li, Liangrong Duan, Tianjiao Zhang, Qi Jia

**Affiliations:** 1 Key Research Institute of Yellow River Civilization and Sustainable Development, Henan University, Kaifeng 475001, China; 2 Collaborative Innovation Center of Yellow River Civilization Provincial Co-Construction, Henan University, Kaifeng 475001, China; 3 School of Management, Tianjin University of Technology, Tianjin 300384, China

**Keywords:** construction land expansion, resource-based cities, driving factors, Geodetector, China

## Abstract

Studying construction land expansion (CLE) characteristics and driving factors in resource-based cities (RBCs) is important to promote efficient land use and maintain ecological equilibrium in RBCs. This study explores the CLE and its driving factors in RBCs. The results indicated that (1) the CLE in RBCs became increasingly obvious, and the number of cities with expansion areas exceeding 20 km^2^ increased from 29 to 86. In RBCs in different regions, CLE in eastern, central, and western regions was obvious, while CLE in the northeast region decelerated. The order of CLE degree at different stages of RBCs was mature, growing, regenerative, and declining. (2) Single factors such as gross domestic product, fixed-asset investment, and secondary industry added value, playing a major role. This differs from the dominant role of population and urbanization in existing research. This occurred because population growth is slow, the urbanization rate is low, population contraction prominently occurs, and economic development exhibits notable path dependence in RBCs. (3) Interaction-factor detection demonstrated that the force of two-factor interaction was greater than that of a single factor, and the interactions of total population with fixed-asset investment and economic development level strongly drove CLE in RBCs.

## 1. Introduction

Construction land (CL) refers to land for residential areas, transportation facilities, special use, mining areas, etc. [1]. Construction land expansion (CLE) indicates other types of land that are continuously occupied by construction land. CLE changes the habitats, biogeochemistry, hydrology, land cover, and surface energy balance [2]. The disorderly CLE has increasingly emerged as a limiting factor of regional sustainable development [3]. As one of the countries with the fastest-growing industrialization and urbanization, China faces dramatic demands for construction land [4,5]. Due to the large-scale CLE, regional socioeconomic and ecological problems, such as the reduction of cultivated land, food shortage, land price inflation, traffic congestion, and environmental pollution, occur frequently [6,7]. Thus, how to effectively control CLE, deal with the socioeconomic and ecological problems caused by CLE, and find the solutions for regional sustainable development, has become an important research concern.

Land use change is a core field of global environmental change and has been a focus for many researchers in past decades [8,9]. CLE is a significant feature of land use change, which consumes cultivated land, ecological land, etc. [9,10,11]; therefore, the CLE has become the focus of numerous studies [12]. Currently, research on CLE mainly focuses on three aspects, namely, the impact of CLE on sustainable development, spatiotemporal evolution characteristics of CLE, and the driving factors of CLE. As to the impact of CLE on sustainable development, scholars explored the negative effects of CLE; such as the decrease in cultivated land [13,14,15], spatial evolution of rural settlements [16], eco-security and biodiversity loss [17,18,19], air pollution [20,21], and the increase in tropospheric ozone concentration [18]. Regarding the spatiotemporal evolution characteristics of CLE, some studies discussed the trend of CLE in the past decades [7], while others predicted the trend of CLE in the future [2,10,22]. The method, such as the sprawl intensity index [23], exponential landscape expansion index [24,25], spatial center shift model, urban land expansion estimation [26], and ArcGIS-based spatial analysis [27], have been mainly used in the analysis of a single city [18], urban agglomeration, or provincial area [27,28,29], all cities in a given nation [30,31] and global scale [10,26]. Concerning the driving factors of CLE, many scholars have proposed that CLE is affected by population growth [32,33], and economic development [34,35,36], while others have indicated that CLE is related to the behavior of local governments [23,37,38]. In general, factors such as population, economic level, output value of the secondary and tertiary industries, industrial structure, fixed-asset investment, actual utilization of foreign capital, and fiscal expenditure [23,27,36,38,39,40], are often employed in CLE driving factors analysis. While discussing the driving factor of CLE, many researchers have taken the methods of rank scale theory, correlation analysis, partial correlation analysis, and econometric models; however, few have been concerned with the impact of factor interactions on CLE.

Resource-based cities (RBCs) are those whose leading industries involve the exploitation and processing of natural resources, such as minerals, forests, and fossil fuels [41,42,43]. As national bases for the supply of energy and raw materials, RBCs have made a notable contribution to national economic development; however, RBCs are also the most typical areas of ecological destruction in China [44,45]. The agglomeration of population and industry brought by resource exploitation in RBCs requires substantive CL as the space carrier, such as mining land, industrial land, oil field, saltworks, quarry, residential land, transportation land, etc. The CLE, to some extent, reduces ecological land, which poses huge challenges to ecosystem stability. Exploring CLE spatial patterns and influencing factors in RBCs plays an important role in improving the efficiency of CL and stabilizing the ecosystem in RBCs. Unfortunately, relevant researches on RBC focus on the following aspects: the mechanism and policy measures of sustainable development [46], the factors or mechanisms influencing transformation, transformation performance and patterns [47,48,49], alternative industry selection [50], development efficiency evaluation [51,52] and vulnerability assessment [51], ecological footprint and ecological carrying capacity [53], social–economic–environmental coupling coordination characteristics [54,55], spatiotemporal patterns of industry and land use [23,56], and classification of RBCs [57].

In summary, it is found that the current research on CLE and RBCs could provide an important reference value; however, there are two gaps. First, although the impact of multiple factors on CLE has been considered, the impact of the interaction among multiple factors on CLE has been ignored. Second, there are few studies on CLE in RBCs, which need to be further expanded. RBCs are widely distributed in China and even the world. The economic development level and policy tendency change in different regions and different development stages of RBCs. It is important to identify the spatiotemporal pattern of CLE in RBCs in various regions and at different development stages to analyze the driving factors. To fill these gaps, this paper adopted 126 prefecture-level RBCs in China as research objects, examined the spatial pattern characteristics of CLE in the RBCs, and studied the driving factors of CLE by taking the Geodetector method from a single element and element interactions perspective.

## 2. Data and Methods

### 2.1. Study Area

**Figure 1:**
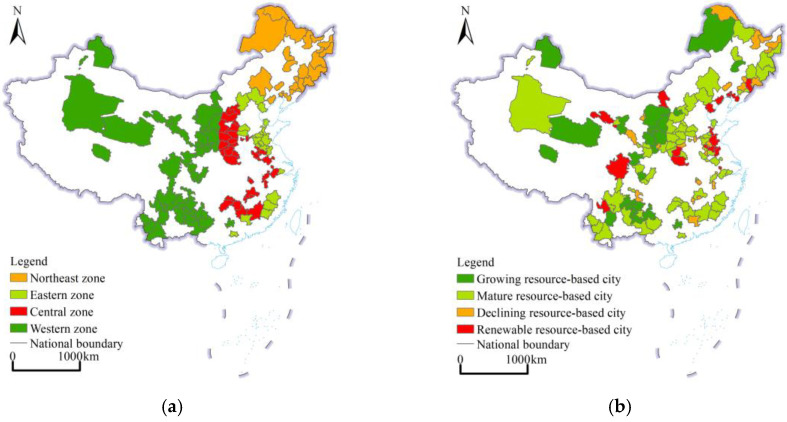
Classification and distribution of RBCs in China. (**a**) Classification of RBCs by region; (**b**) classification of RBCs by development stage.

In the national sustainable development plan for RBCs (2013–2020) issued by the State Council, 262 RBCs were identified in China, including 126 prefecture-level cities, 62 county-level cities, 58 counties (autonomous counties and forest areas), and 16 municipal districts (development and management zones) [41,42,43]. In this study, 126 prefecture-level cities were chosen as the research objects (Figure 1a). According to the different regions, among the 126 RBCs, the western region contains the most RBCs (46 cities), accounting for 36.5% of the total number of prefecture-level RBC in China; there are 37 RBCs in the central region, accounting for 29.4% of the total number of RBCs; and 23 RBCs in the northeastern region and 20 in the eastern region, accounting for 18.2% and 15.9%, respectively, of the total number of RBCs in China (Figure 1a). According to the above plan, RBCs can be divided into four types, namely, growing, mature, declining, and regenerative types, according to the resource security ability and sustainable economic and social development capacity; among these types, the numbers of growing, mature, declining, and regenerative cities are 20, 66, 24 and 16, respectively (Figure 1b). By 2018, 126 RBCs exhibited a construction area of 85,000 km^2^, accounting for 31.71% of the total CL area in China, the total population reached 440 million people, accounting for 31.84% of the total population in China, and the gross domestic product (GDP) amounted to 20,180.2 billion yuan, accounting for 22.41% of the national GDP.

### 2.2. Data

This study was conducted at the city scale, and the time span was 1995–2018, of which the period from 1995 to 2015 was divided into 5-year intervals; since some data for 2020 were unavailable, the latest data were obtained for 2018, thereby ensuring that all data could be synchronously obtained. Therefore, 2015–2018 was considered a separate period. The data used in this paper included the following: (1) land use/cover changes (LUCCs) data were obtained from the Land Use/Land Cover Remote Sensing Detection Database for 1995, 2000, 2005, 2010, 2015, and 2018, with a spatial resolution of 30 m × 30 m. The first-level land classification includes 1—cultivated land, 2—forestland, 3—grassland, 4—water area, 5—CL, which includes urban land (build up area), rural residential land, and other construction land (mining land, industrial land, oil field, saltworks, quarry, transportation land, airport, and special land), and 6—unused land, etc. These data were used to extract the CL area in each RBC. The data were obtained from the Resource and Environmental Science and Data Center (https://www.resdc.cn/ (accessed on 10 October 2021)) of the Institute of Geographical Sciences and Natural Resources Research, Chinese Academy of Sciences. (2) National, provincial and municipal boundary vector data were used to extract the boundaries of each city. The data originated from 1:1 million data provided by the National Basic Geographic Information System. (3) In the analysis of influencing factors, the socioeconomic data involved in the indicators were acquired from the *China Urban Statistical Yearbook* from 2001 to 2019, *Statistical Yearbook of Provinces* (cities and autonomous regions), *Statistical Bulletin of National Economic and Social Development of Cities* from 2000 to 2018, and *China Land Resources Statistics Yearbook*.

### 2.3. Methods

**Figure 2:**
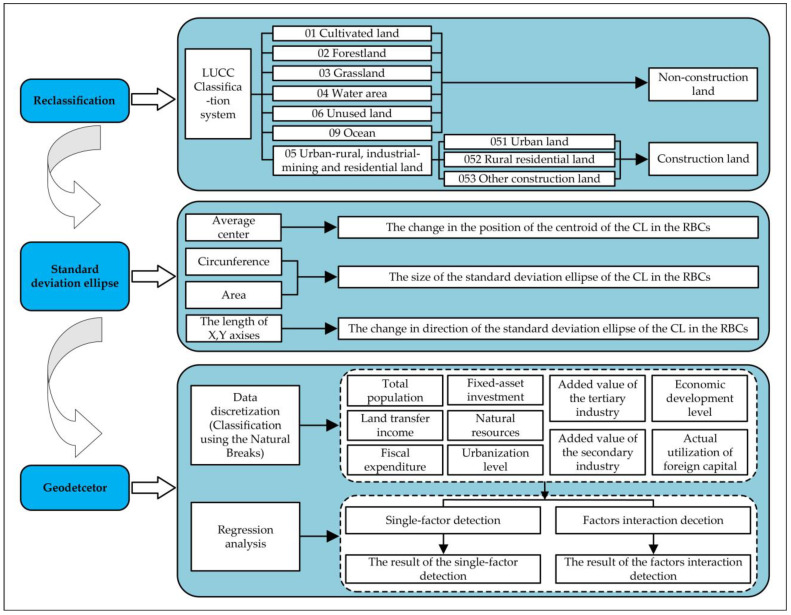
The flowchart of methods.

Based on LUCCs data, this paper uses reclassification technology to extract the CL of RBCs, scrutinizes the spatiotemporal differentiation characteristics of CLE by taking the standard deviation ellipse, and analyzes the driving factors of CLE in RBCs by employing the geographic detector. The flow of various methods is shown in Figure 2.

#### 2.3.1. Reclassification

One of the important functions of ArcGIS is the determination of potential information via classification. According to different research needs, the original data can be reclassified and extracted in ArcGIS 10.8 software (ESRI, Redlands, CA, USA) to obtain the required data. This process is referred to as reclassification. The main purposes of reclassification are as follows: (1) old values are replaced with new values; (2) multiple sets of values are grouped into one class; (3) the values of a group of grids are reclassified according to the same level; and (4) specified values are set to null or null values are set to a certain value. This study used the reclassification method to extract CL in 126 RBCs from the obtained LUCCs data. There are six first-class land types in the LUCCs data. According to the LUCCs classification system, mining land, industrial land, oil field, saltworks, quarry, residential land, transportation land, airport, and special land were categorized as CL types.

#### 2.3.2. Standard Deviation Ellipse

The standard deviation ellipse method was proposed by D. Welty Lefever, a professor at the University of Southern California, in 1926. This method can be used to analyze the spatial distribution direction and trend of geographic elements, and reveal the overall characteristics of the spatial distribution of geographic elements. The standard deviation ellipse method includes parameters, such as the center of the ellipse, azimuth angle, and long and short axes. This method has been widely used in the study of economic spatial patterns and terrain distributions. In this study, the center of the ellipse was used to analyze the average center of CLE in RBCs.

The azimuth represents the angle formed by the true north of the ellipse and the clockwise long axis, while the direction and length of the two axes could be used for RBC analysis. The directional characteristics and dispersion degree of CLE can be calculated as follows in Equation (1). The direction and length of two axes are used to analyze the directional characteristics and discrete degree of construction land expansion in RBCs. The related calculation formulas are as follows:

The mean center of the standard deviation ellipse (${\bar{X}}_{w}$,${\bar{Y}}_{w}$) can be obtained as:

(1) $${\bar{X}}_{w}=\frac{\sum_{i=1}^{n}w_{i}x_{i}}{\sum_{i=1}^{n}w_{i}},{\bar{Y}}_{w}=\frac{\sum_{i=1}^{n}w_{i}y_{i}}{\sum_{i=1}^{n}w_{i}}$$

The azimuth is α can be calculated as:

(2) $$\;\tan\alpha =\frac{\begin{array}{c} \;\;\;\;\left(\sum_{i=1}^{n}w_{i}^{2}{\tilde{x}}_{i}^{2}-\sum_{i=1}^{n}w_{i}^{2}{\tilde{y}}_{i}^{2}\right)+\sqrt{{\left(\sum_{i=1}^{n}w_{i}^{2}{\tilde{x}}_{i}^{2}-\sum_{1}^{n}w_{i}^{2}{\tilde{y}}_{i}^{2}\right)}^{2}+4\sum_{i=1}^{n}w_{i}^{2}{\tilde{x}}_{i}^{2}{\tilde{y}}_{i}^{2}w_{i}^{2}{\tilde{x}}_{i}^{2}{\tilde{y}}_{i}^{2}} \end{array}}{2\sum_{i=1}^{n}w_{i}^{2}{\tilde{x}}_{i}{\tilde{y}}_{i}}.$$

The x-axis standard deviation $\sigma_{x}$ and y-axis standard deviation $\sigma_{y}$ can be determined as:

(3) $$\sigma_{x}=\sqrt{\frac{\sum_{i=1}^{n}{\left(w_{i}{\tilde{x}}_{i}\cos\alpha -w_{i}{\tilde{y}}_{i}\sin\alpha \right)}^{2}}{\sum_{i=1}^{n}w_{i}^{2}}}$$

(4) $$\sigma_{y}=\sqrt{\frac{\sum_{i=1}^{n}{\left(w_{i}{\tilde{x}}_{i}\sin\alpha -w_{i}{\tilde{y}}_{i}\cos\alpha \right)}^{2}}{\sum_{i=1}^{n}w_{i}^{2}}}$$

where ($x_{i}$,$y_{i}$) denotes the spatial location of the RBC, $w_{i}$ denotes the corresponding weight, and (${\tilde{x}}_{i}$,${\tilde{y}}_{i}$) denotes the coordinate deviation from the spatial location of each RBC to the average center (${\bar{X}}_{w}$,${\bar{Y}}_{w}$).

#### 2.3.3. Geodetector

The Geodetector is a new spatial statistical method used to detect the spatial differentiation of elements and reveal the driving factors behind this process [58], which has been widely employed in the study of the impact mechanism of the natural environment and social economy. The Geodetector can not only detect numerical data but can also detect the impact of the interaction between two factors on the dependent variable, which is an effective tool to study the driving effect of complex geographic factors. When processing the original data, this study drew lessons from the existing research to discretize and layer the independent variable data by using the natural breakpoint method. As such, in this study, factor differentiation and interaction detection were performed, thereby identifying the driving factors of CLE in RBCs and their interactions and examining the main influencing factors of CLE and their impact degree after controlling for the interaction among various factors.

Differentiation and factor detection: the differentiation in Y and the extent to which a certain factor X can explain the spatial differentiation in attribute Y were determined, where Y denotes the CL type in a given RBC, and X is the driving factor. The equation is as follows:

(5) $$q=1-\frac{1}{{N\sigma }^{2}}\overset{L}{\underset{h=1}{\sum }}N_{h}\sigma_{h}^{2}$$

where q is the influence detection index of the driving factors of CL in the RBC, N is the number of samples in the entire region, i.e., 126 RBCs, which is the number of sample units in the partition area, h = 1, 2, 3, …, L denotes the stratification of variable or a given factor, is the variance in the dependent variable within the entire region, and is the variance within the partition area. The value range of q is [0, 1]. For q = 0, the observed factors do not drive CLE in the considered RBCs. The larger the value of q is, the greater the driving effect of the observed factors on CLE in the RBCs.

Interaction detection: in this study, this technique was used to identify the interaction between different driving factors and evaluate whether the combined action of two driving factors enhanced or reduced the explanatory power of the dependent variable Y, e.g., factors A and B influencing the change in CL in RBCs. A new layer C could be obtained by spatially stacking layers A and B, and the attributes of C were jointly determined by those of A and B. By comparing the influence of the factors in layers A and B to the influence of the factors in layer C, we could determine whether the interaction between two factors on CL in RBCs was greater or less than the influence of a single factor.

Interactive detection occurs as follows: q(A∩B) < min(q(A),q(B)) indicates that the nonlinearity is reduced after interaction between factors A and B; min(q(A),q(B)) < q(A∩B) < max(q(A),q(B)) indicates that the single-factor nonlinearity is reduced after interaction between A and B; q(A∩B) > max(q(A),q(B)) indicates that the two factors mutually increase after interaction; q(A∩B) > q(A) + q(B) indicates that the two factors enhance each other in a nonlinear manner; and q(A∩B) = q (A) + q(B) indicates that the two factors are independent.

## 3. Results

### 3.1. Spatiotemporal Evolution Characteristics of Construction Land

#### 3.1.1. General Characteristics

**Figure 3:**
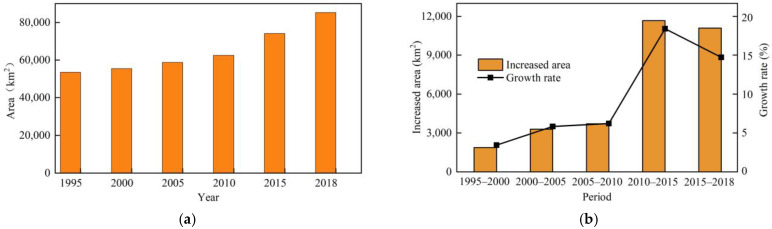
Change status of construction land in RBCs. (**a**) Construction land area; (**b**) growth rate of construction land.

ArcGIS software was used to reclassify the LUCCs data and extract the CL area in RBCs. Statistics revealed that the CL area greatly changed from 1995 to 2018 (Figure 3a), in which the changes from 2010–2015 and 2015–2018 were very obvious (Figure 3b). From 1995 to 2018, the CL area increased from 53,590.04 to 85,224.76 km^2^. Adopting 1995 as the base year, the growth rate of the CL area in 2018 reached 59.03%. During the study period, the CL growth rate exhibited an increasing trend from 1995 to 2015. From 2010 to 2015, the CL growth rate reached up to 18.67% and the CL area increased by 11,664.47 km^2^, while it decreased from 2015 to 2018 because after 2015, to adapt to the new normal of China’s economy, China has continuously strengthened the protection of cultivated land and the intensive use of land resources [59]. The government has issued a series of land policies, such as Guiding Opinions on Further Promoting the Redevelopment of Inefficient Urban Land (Trial Implementation) [60], National Land Planning Outline (2016–2030) [61], and National Land Improvement Plan (2016–2020) [62]. These policies have effectively reinforced the intensive and economical use of land resources, which led to a decline in the growth rate.

**Figure 4:**
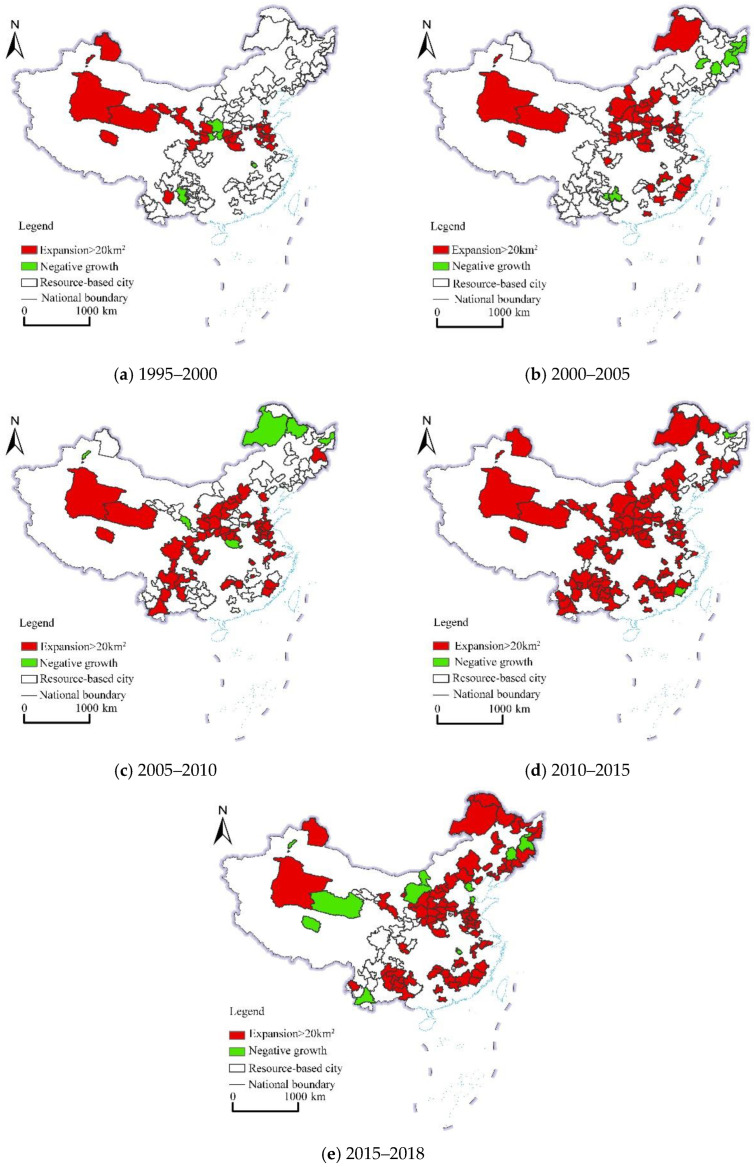
Spatial pattern of construction land change in RBCs (1995–2018).

In terms of different regions, CL was mainly distributed in the eastern and central regions, whereas relatively few CL areas were observed in the western and northeastern regions. As shown in Figure 4, during the study period, among the four regions, the eastern, central, and northeastern RBCs exhibited significantly expanded CL areas. From 1995 to 2000, there were 29 cities whose CL area increased by more than 20 km^2^, such as the Haixi Mongolian and Tibetan Autonomous Prefecture and Jiaozuo. Only five cities were observed whose CL area decreased during the same period, namely, Huangshi, Weinan, Yan’an Xianyang, and Qujing. From 2000 to 2005, there were 48 cities whose CL area increased by more than 20 km^2^, such as Longyan, Tangshan, and Dongying. During the same period, there were nine cities with a reduced CL area, such as Qitaihe, Jixi, and Mudanjiang. From 2005 to 2010, 59 cities were observed with an increase of more than 20 km^2^ in their CL area, such as Yulin, Ezhou, and Huangshi. During the same period, seven cities occurred with a decrease in their CL area, including Baiyin, Shuangyashan, and Karamay. From 2010 to 2015, there were 101 cities with an increase of more than 20 km^2^ in their CL area, such as Zhangjiakou and Yulin, and only 2 cities with a decrease in the CL area were observed during the same period, namely, Longyan and Hegang. From 2015 to 2018, there were 86 cities with an increase of more than 20 km^2^ in the CL area, including Linyi, Ganzhou, and Luliang, while 13 cities with a decrease in the CL area were observed during the same period, such as Baotou, Wuhai, and Pu’er. In the study area, from 1995 to 2018, there were 9 cities with an increase of more than 20 km^2^ in their CL area, such as Jining, Puyang, and Yuncheng. During the study period, the number of cities with a CL increase exceeding 20 km^2^ continued to increase, indicating that CLE obviously occurred in these RBCs.

#### 3.1.2. Regional Characteristics

**Figure 5:**
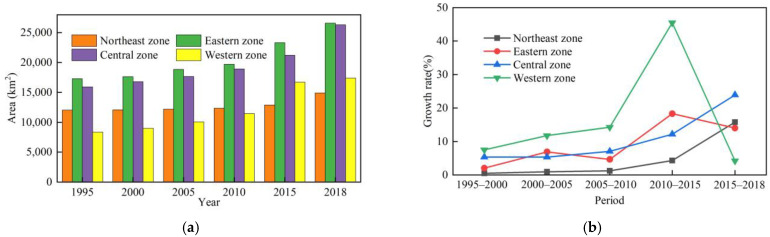
Change in construction land in the RBCs in the different regions. (**a**) Construction land area; (**b**) growth rate of construction land.

The CL area in the RBCs in the four regions exhibited an upward trend (Figure 5a). During the study period, the CL area in the RBCs was similar between the eastern and central regions, and these two regions experienced the largest expansion in the CL area among the four regions. The CL area in the RBCs in the western region increased approximately 2 times, and after 2015, the area exceeded that in northeast region, which was mainly related to the 2 rounds of western development policies implemented by the state since 2000. The CL area in the RBCs in the northeastern region still increased, but the extent was limited because the RBCs in the northeastern region lacked growth momentum, the population growth rate declined, and the demand for CL decelerated.

From the perspective of the CL growth rate, from 1995 to 2018, the CL change status in the RBCs in each region was as follows (Figure 5b): (1) the CL growth rate in the RBCs in the eastern region indicated a fluctuating upward trend, of which the highest growth rate of 18.33% from 2010–2015 occurred because the eastern region exhibited the fastest economic growth and highest economic vitality in China. In the process of rapid economic growth, it was highly necessary to increase the CL area to ensure production and living activities, so the CL area in the RBCs in this region rapidly expanded. (2) The CL growth rate in the RBCs in the central region consistently revealed an increasing trend, and the growth rate was the highest from 2015 to 2018, at 23.93%, indicating that with the implementation of strategies such as the Rise of Central China and Central Plains Urban Agglomeration, the RBCs in the central region could achieve more adequate development. With the large population in the central region, the demand for CL was high, so the CL expansion intensity and speed were accelerated. (3) The CL area in western RBCs was the smallest among the four regions, with a total increase of 9051.09 km^2^. The growth rate was the highest between 2010 and 2015, at 45.48%. From 2015–2018, the growth rate sharply declined, indicating that the expansion intensity decreased. The RBCs in the western region were characterized by a small CL area and a relatively low economic development level, so the expansion was not notable. (4) Before 2010, the CL area in the RBCs in the northeast region slowly increased, with a growth rate lower than 2% during each period. After 2010, the trend of CL expansion accelerated, especially between 2015 and 2018, and the growth rate increased to 15.76%.

#### 3.1.3. Characteristics of the Development Stages

**Figure 6:**
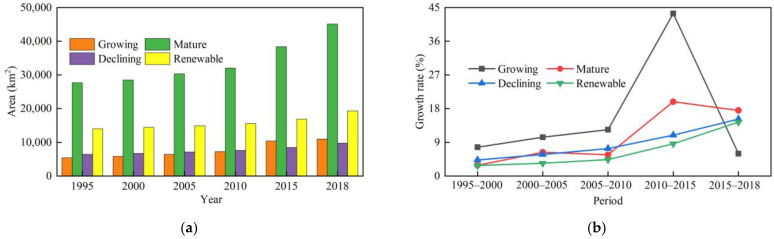
The change of construction land of RBC at various development stages. (**a**) Construction land area; (**b**) growth rate of construction land.

The CL area in the various RBCs at the different development stages continuously expanded (Figure 6a), but there was a gap in the expansion area. The order of the CLE area was mature-type RBCs > growing-type RBCs > regenerative-type RBCs > declining-type RBCs. Among these four types, the total amount of CL in the mature RBCs was always larger than that in the other three types of RBCs, with the greatest increase in area representing a total increase of 17,429.07 km^2^. In regard to CLE in mature RBCs, on the one hand, the process benefited from the large number of mature RBCs, namely, 66 cities, accounting for 52.38% of the total number of cities (126); on the other hand, the economic development process in these cities occurred at the stage of rapid improvement, in which it was urgent to extend the industrial chain and construct industrial clusters, resulting in a high demand for CL. However, the resources in declining cities were increasingly exhausted, the development momentum was insufficient, these cities gradually declined, and population growth occurred slowly or the population even decreased (data from the Seventh National Census), so the CLE phenomenon was accordingly decelerated.

Based on the CL growth rate, the change in CL in the various types of RBCs from 1995 to 2018 was as follows (Figure 6b): (1) the CL area in the growing RBCs increased by 5576.95 km^2^, which is consistent with the overall CL growth rate in the 126 RBCs, indicating an upward trend from 1995 to 2015, with the fastest growth from 2010 to 2015, at 43.42%, and a downward trend after 2015. (2) The CL area in the mature RBCs increased by 17,429.07 km^2^, the growth rate reached peaked (19.82%) from 2010 to 2015, and then slightly declined. (3) The CL area in the declining RBCs was the smallest among the four regions, with a total increase of 3301.65 km^2^, but the growth rate was increasing. (4) The CL growth rate in the regenerative RBCs was not high, with a total increase of 5327.05 km^2^, but the growth rate was also persistently increasing.

### 3.2. Spatial Concentration Degree and Direction Changes in Construction Land

**Figure 7:**
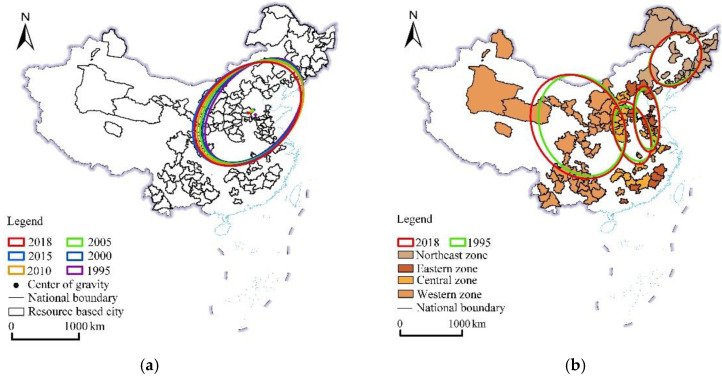
Standard deviation ellipse results for construction land in RBCs (1995–2018). (**a**) Standard deviation ellipse; (**b**) standard deviation ellipse in different regions.

With the use of ArcGIS software and application of the CL area in each city as the weight, the standard deviation ellipse of the CL change in the RBCs from 1995 to 2018 (Figure 7a) was obtained, and the relevant parameters are listed in Table 1. Figure 7a shows that the CL area in the RBCs exhibited a trend of expanding from northeast to southwest. From 1995 to 2015, the distribution range of the standard deviation ellipse of CL in the RBCs gradually expanded; however, from 2015 to 2018, the distribution range of the standard deviation ellipse contracted, indicating that CLE in the RBCs was clustered in space. The barycenter coordinates moved from (116°16′ E, 36°41′ N) to (114°96′ E, 36°78′ N), and the moving distance reached 115 km^2^. Overall, the azimuth angle increased, and the short axis of the standard deviation ellipse increased, indicating that the main driving force of CLE in the RBCs originated from the east–west direction. This finding could be explained as follows: from 1995 to 2018, the RBCs in the eastern, central, and western regions achieved a relatively high intensity of land development, the average land development intensity increased by 4.04%, 2.86%, and 1.08%, respectively, and the CL area significantly expanded. During the same period, the land development intensity in the RBCs in the northeast region only increased by less than 1%, and CLE was not obvious (Table 2).

To analyze the variation characteristics of CLE in the RBCs in detail, standard deviation ellipses were generated for each region, and the following results could be obtained: from 1995 to 2018, (1) CLE in the RBCs in Northeast China was not notable, mainly due to the lack of growth power in the RBCs within this region, and the demand for CL declined. With increasing land development intensity in southern cities such as Huludao, Panjin, and Anshan, and decreasing land development intensity in northeastern cities such as Mudanjiang and Qitaihe, the standard deviation ellipse slightly expanded from north to south. (2) The expansion direction of the CL area in the RBCs in the eastern region was the north–south direction. The RBCs in this region were mainly distributed in Hebei, Shandong, and Jiangsu provinces. The number of RBCs in Zhejiang, Fujian, and Guangdong provinces was small, and the development intensity was low. The driving force of spatial expansion in this region mainly originated from Hebei, Shandong, and Jiangsu provinces. From the perspective of the land development intensity, the land development intensity in both northern cities, such as Zhangjiakou, Zibo, Dongying, and Laiwu, and southern cities, such as Suqian and Xuzhou, increased, so the standard deviation ellipse expanded along the north–south direction. (3) The expansion direction of CL in the RBCs in Central China was the north–south direction, and the standard deviation ellipse range increased, indicating that the urban CL area significantly increased along the north–south direction. From the perspective of the land development intensity, the land development intensity in eastern and western cities decreased, such as Huainan, Maanshan, and Bozhou, but the land development intensity in northern and southern cities increased, such as Yangquan, Lvliang, Ezhou, and Yichun, so the standard deviation ellipse expanded along the north–south direction. (4) The expansion direction of CL in the western RBCs was the east–west direction, and the expansion was very obvious. In this area, only the land development intensity in Baotou city decreased, whereas the land development intensity in the other cities exhibited an increasing trend, especially in the RBCs in the southwestern region such as Zhaotong, Bijie, Qiannan, and Qianxinan, which promoted westward expansion of the standard deviation ellipse.

To analyze the variation characteristics of ECL in the RBCs in detail, standard deviation ellipses were generated for each region, and the following results could be obtained: from 1995 to 2018, (1) ECL in the RBCs in Northeast China was not notable, mainly due to the lack of growth power in the RBCs within this region, and the demand for CL declined. With increasing land development intensity in southern cities such as Huludao, Panjin, and Anshan, and decreasing land development intensity in northeastern cities such as Mudanjiang and Qitaihe, the standard deviation ellipse slightly expanded from north to south. (2) The expansion direction of the CL area in the RBCs in the eastern region was the north–south direction. The RBCs in this region were mainly distributed in Hebei, Shandong, and Jiangsu provinces. The number of RBCs in Zhejiang, Fujian, and Guangdong provinces was small, and the development intensity was low. The driving force of spatial expansion in this region mainly originated from Hebei, Shandong, and Jiangsu provinces. From the perspective of the land development intensity, the land development intensity in both northern cities, such as Zhangjiakou, Zibo, Dongying, and Laiwu, and southern cities, such as Suqian and Xuzhou, increased, so the standard deviation ellipse expanded along the north–south direction. (3) The expansion direction of CL in the RBCs in Central China was the north–south direction, and the standard deviation ellipse range increased, indicating that the urban CL area significantly increased along the north–south direction. From the perspective of the land development intensity, the land development intensity in eastern and western cities decreased, such as Huainan, Maanshan, and Bozhou, but the land development intensity in northern and southern cities increased, such as Yangquan, Lvliang, Ezhou, and Yichun, so the standard deviation ellipse expanded along the north–south direction. (4) The expansion direction of CL in the western RBCs was the east–west direction, and the expansion was very obvious. In this area, only the land development intensity in Baotou city decreased, whereas the land development intensity in the other cities exhibited an increasing trend, especially in the RBCs in the southwestern region such as Zhaotong, Bijie, Qiannan, and Qianxinan, which promoted westward expansion of the standard deviation ellipse.

### 3.3. Analysis of the Driving Factors of CLE

A large number of empirical studies have demonstrated that population growth, economic development, and urbanization affect CLE. Referring to relevant achievements [23,27,36,38,39,63], this study observed the influences of the total population, urbanization level, economic development level, fixed-asset investment, fiscal expenditure, actual utilization of foreign capital, land transfer income, added value of the secondary and tertiary industries, and resource endowment on CLE (Table 3). Among these factors, resource endowment refers to the research of Chen Jianbao [64] and Li Hong [65], which can be represented by the number of employees in the mining industry. Among the 126 RBCs, the CL area in a small number of RBCs exhibited negative growth, which were not used as observation objects. Due to the different establishment times of the various RBCs, to ensure data continuity, single driving factors and the interaction between two factors of CLE in the RBCs in 2005, 2010, 2015, and 2018 were examined.

#### 3.3.1. Single-Factor Detection Results

Single factors were detected via the Geodetector, and the results demonstrated that the total population, economic development level, fixed-asset investment, fiscal expenditure, and added value of the secondary industry all passed the significance test at the level of 1%; urbanization level (2015), actual utilization of foreign capital and land transfer income (2010–2018), added value of the tertiary industry (2005–2015), and natural resources (2005–2010) passed the significance test at the 5% level (Table 4).

In terms of the explanatory power of a single factor, from 2005 to 2015, the economic development level, added value of the secondary industry, added value of the tertiary industry, and fixed-asset investment were the main reasons for CLE in the RBCs. From 2015 to 2018, the economic development level, added value of the secondary industry, and fixed-asset investment were the main reasons for CLE in the RBCs. The above results indicated that CLE was closely related to the economic level and that economic development drove the CLE process.

In existing studies [23,27,36,38,39], the population and urbanization level obviously affect CLE, but this study revealed that the impact of the population on CLE in the RBCs was limited, and the urbanization level failed to pass the significance test in most years. This occurred because of the notable path dependence of population growth and economic development in the RBCs. During the study period, the population growth and urbanization rates in the RBCs were lower than those in all cities and non-resource-based cities (NRBCs) in China (Table 5). There were 37 RBCs with population shrinkage, while there were 58 NRBCs exhibiting population decline, accounting for 29.60% and 24.58%, respectively, of the total number of two types of cities, reflecting that the phenomenon of population decline in the RBCs was highly notable. Therefore, in the RBCs, slow population growth, a low urbanization rate, and population shrinkage resulted in a lower demand for CL. In addition, the RBCs established specific industries closely related to resource-based industries, such as mining, industry and mining, and manufacturing, and economic growth depended on scale expansion of these industries. Therefore, a large number of other CL types (factory and mining land, large industrial land, oilfields, salt fields, quarries, etc.) were required as space carriers, which could be verified from the structural characteristics of the various CL types in the RBCs. In the RBCs, the expansion rate of the other CL types was higher than that of urban and rural residential land (Table 6).

Overall, the economy and investment obviously affected CLE in RBCs. The influence of economic factors was mainly manifested in the fact that the economic development level in RBCs mainly depended on extractive, mining, manufacturing, and other industries requiring a large amount of industrial and mining land for support, so economic growth drove CLE. Fixed-asset investment is often used in municipal public facilities and real estate; more importantly, it is used in the construction of industrial infrastructure, such as that in extractive, mining, and manufacturing industries, thereby promoting an increase in the CL scale. To a certain extent, land transfer, fiscal expenditure, and fixed-asset investment reflect the government policy tendency. The government expropriates agricultural land at a low price and sells it at a high price. Various investment and construction behaviors of developers after land purchase also promote CLE.

#### 3.3.2. Factor Interaction Detection Results

Based on single-factor analysis, considering the influence of factor interactions on CLE in the RBCs, factor interaction detection was further used for analysis. The factor interaction detection results (Table 7) revealed that the relationship was enhanced after factor interaction, indicating that two-factor interaction could enhance the interpretation of CLE. Among them, in 2005, the interaction between the total population (X_1_) and fixed-asset investment (X_4_), economic development level (X_3_), added value of the secondary industry (X_8_), fiscal expenditure (X_5_), and added value of the tertiary industry (X_9_) was notable, at 0.73, 0.65, 0.62, 0.62, and 0.57, respectively. In 2018, fixed-asset investment (X_4_) and actual utilization of foreign capital (X_6_), total population (X_1_) and fixed-asset investment (X_4_), total population (X_1_) and economic development level (X_3_), economic development level (X_3_) and fixed-asset investment (X_4_), and fixed-asset investment (X_4_) and fiscal expenditure (X_5_) exhibited obvious interactions, at 0.69, 0.65, 0.63, 0.62, and 0.62, respectively. From 2005 to 2018, the interaction between the total population, fixed-asset investment, and economic development level was strong. Each of these three indicators notably impacted CLE. After the interaction between any two factors, the impact on CLE further increased. This suggests that with the support of the population, the economic development and investment levels could be improved, fixed-asset investment could be increased, infrastructure could be optimized, the urban development rate could be enhanced, employment opportunities could be increased, more people could be attracted to gather, and the demand for land could be expedited, thus promoting CLE to a certain extent.

## 4. Discussion and Conclusions

### 4.1. Discussion

Based on the above results of this study, the following points should be considered:

First, according to the basic data of CLE research, existing studies mostly use statistical and nighttime light data, which cannot effectively support systematic research on CLE in RBCs. Regarding statistical data, due to the influence of factors such as the RBC establishment time and adjustment of administrative divisions, it is difficult to obtain comprehensive data pertaining to CL in the 126 prefecture-level RBCs in China, and there are certain limitations; moreover, nighttime light data are not real land use data and can only represent the CL status to a certain extent, thus indicating certain limitations. The LUCCs data used in this study could provide CL data considering the primary LUCCs classification system based on the boundaries of the 126 RBCs in China and the use of ArcGIS reclassification technology, which could be used on large scales. To a certain extent, this approach could overcome the limitations of statistical and nighttime light data.

Second, studies have demonstrated [23,27,36,38,39] that the number of permanent residents, urbanization level, and economic development level play a leading role in CL expansion, while the influence of fixed-asset investment is limited. However, this study demonstrated that in the considered RBCs, the population factor slightly influenced CLE, whereas the economic development level, fixed-asset investment, and added value of the secondary industry played a leading role in CLE. The reasons were related to the slow population growth, low urbanization rate, and notable path dependence of economic development in the RBCs: on the one hand, slow population growth, a low urbanization rate and highly obvious population contraction in the RBCs resulted in low demand for CL among urban and rural residents; on the other hand, economic development in the RBCs exhibited notable path dependence. Economic development mainly relied on secondary industries closely related to resource industries, such as the extractive, industrial mining, and manufacturing industries. Economic growth thus depended on the expansion of the scale of these industries. Therefore, a large number of other CL types (factory and mining areas, large industrial areas, oilfields, salt fields, quarries, etc.) were required as space carriers; fixed-asset investment is often used for municipal public facilities and real estate development. More importantly, it is used in the construction of industrial infrastructure, such as that in the extraction, mining, and manufacturing industries, thereby promoting an increase in the CL scale. In addition, in previous studies [66], when the study area contained numerous RBCs, the degree of influence of various driving factors on CLE was similar to that determined in this study.

Third, considering the results of this study, the CLE phenomenon was very prominent in the RBCs, CL was extensively developed, and its intensive utilization space was large. During the research period, among the 126 RBCs in China, the number of cities with the CLE amount exceeding 20 km^2^ was large, reflecting the high demand for production and living areas in these RBCs over the past 25 years, which imposed notable pressure on the agricultural production space and ecological space [67]. In green transformation and development of RBCs in the future, the scale, timing, and intensity of resource extraction should be reasonably regulated; the boundaries of urban development, the red line of arable land protection, and the red line of ecological protection should be scientifically delineated [68]; the mode of economic development should be transformed; industrial structure upgrading should be accelerated; rational allocation of population, industry, and production factors should be guided; the efficiency and intensive use of CL should be improved; control of the land use space should be strengthened; and the RBC development intensity should be reduced. Around 2015, China launched a series of land policies including Promoting the Redevelopment of Inefficient Urban Land (Trial Implementation) [60], National Land Planning Outline (2016–2030) [61], and National Land Improvement Plan (2016–2020) [62], which have effectively controlled the CLE in RBCs (it is verified by the decline in the growth rate of CL in RBCs after 2015); these policies have also played an active role in farmland and ecological space protection. Thus, these policies should be continuously implemented in the future.

Fourth, there exist differences between this and existing studies in terms of driving factor analysis conclusions, which may be attributed to the following: (1) there are differences in the temporal and spatial study scales, e.g., certain studies focused on CLE at the national [24], urban agglomeration [9], and provincial [27] scales, while other studies focused on CLE during different periods (e.g., 1990–2018 [69], 2000–2015 [23], and 2006–2015 [24]). This study focused on the driving factors of CLE in 126 RBCs in China from 2005 to 2018. Therefore, the different temporal and spatial research scales could lead to differences in the obtained driver analysis conclusions. (2) The various studies defined CL differently. Some studies classified urban built-up areas or urban areas [36] as CL areas, while other studies [27,69] uniformly defined urban and rural CL areas, rural homesteads, industrial and mining CL areas, etc., as CL. (3) RBCs and NRBCs exhibit distinct resource backgrounds, industrial structures, etc., and their development orientations and paths vary. Different city types may also lead to differences in the driving factors of CLE.

Fifth, concerning analyzing the driving factors for CLE in RBCs, this study takes economic factors into consideration. Since CLE is affected by a variety of factors and there is interaction between the factors, in future research, natural factors, policy factors, and traffic factors should also be included in the analysis framework of the driving factors for CLE in RBCs to carry out more in-depth research.

### 4.2. Conclusions

By reclassifying LUCCs data for 126 RBCs from 1995 to 2018, this paper examined the spatiotemporal pattern of CLE in these RBCs, employed single-factor and factor interaction detection in the Geodetector, and analyzed the driving degree of different factors on CLE in the considered RBCs. The conclusions are as follows:

(1) From 1995 to 2018, CLE in the RBCs was prominent, and the expansion trend gradually increased. The number of cities with an expansion exceeding 20 km^2^ was large, from 29 to 86. The RBCs in the eastern and central regions exhibited a large CL area and rapidly expanded; in the western region, the CL area was small and expanded relatively slowly; and due to the depletion of resources and loss of population, CL expansion occurred the slowest in the northeastern region. From the perspective of the development stage, the CL area in the various RBCs at the different stages continuously expanded overall, but there was a gap in the expansion rate. The degree of CL expansion could be ranked as mature, growing, regenerative, and declining RBCs.

(2) Standard deviation ellipse analysis revealed that CLE in the RBCs exhibited northeast–southwest evolutionary characteristics. Among the RBCs in the four regions, the expansion process in the northeastern region decelerated, while expansion in the eastern, central, and western regions was obvious. In terms of subregions, the CLE range in the RBCs in the northeastern region expanded to the north and south, and the driving force mainly originated from southern cities such as Huludao, Panjin, and Anshan. The expansion direction in the eastern and central regions was the north–south direction, and the driving force of spatial expansion in the eastern region mainly originated from Hebei, Shandong, and some cities in Jiangsu province. The driving force of spatial expansion in the central region originated from cities such as Yangquan, Luliang, Ezhou, and Yichun. The CL expansion direction in the western region was the east–west direction, and the driving force in the southwestern region largely originated from cities such as Zhaotong, Bijie, Qiannan, and Qianxinan.

(3) The Geodetector analysis (via single-factor detection) determined that the economic development level, fixed-asset investment, and added value of the secondary industry strongly drove CLE in RBCs, reflecting that the economic scale and investment level in RBCs greatly impacted CL expansion; fiscal expenditure and the added value of the tertiary industry notably promoted CLE in most years, indicating that the upgrading of capital and industrial structure also drove CL expansion. In factor interaction detection, factors were enhanced after interaction. The driving force of single-factor pairwise interaction on CLE in RBCs was higher than that of any individual factor, especially the interaction between the total population, fixed-asset investment, and economic development level, which was notable, suggesting that the population (potential labor force) generated a notable interaction effect. Under this support, improvement in the economic development and investment levels could accelerate urban development and promote CL expansion to a certain extent.

## Figures and Tables

**Table 1:** Standard deviation ellipse analysis results.

Years	Longitude of Barycenter	Latitude of Barycenter	Azimuth Angle	Area (km²)	Perimeter (km)
1995	116°16′ N	36°41′ E	32°23′	2,031,245.41	5292.25
2000	115°95′ N	37°21′ E	33°86′	2,104,505.38	5343.40
2005	115°72′ N	37°09′ E	34°28′	2,192,195.44	5415.20
2010	115°44′ N	36°95′ E	35°39′	2,260,801.96	5482.58
2015	114°79′ N	36°83′ E	37°19′	2,331,506.61	5542.20
2018	114°96′ N	36°78′ E	35°41′	2,298,016.82	5503.07

**Table 2:** Average value of the land development intensity in the RBCs in the different regions (%).

Region	1995	2018	1995–2018
Northeastern region	2.97	3.78	0.81
Eastern region	8.50	12.54	4.04
Central region	5.18	8.04	2.86
Western region	1.27	2.35	1.08

**Table 3:** Observation index of driving factor analysis of CLE in RBCs.

Variable	Symbol	Explanation
Construction land area	Y	Construction land area in the city
Total population	X_1_	Total population of the city
Urbanization level	X_2_	Urban population/total population of the city
Economic development level	X_3_	Gross domestic product of the city
Fixed-asset investment	X_4_	Total investment in the fixed assets of the city
Fiscal expenditure	X_5_	The sum of all financial expenditures
Actual utilization of foreign capital	X_6_	Total amount of foreign capital actually utilized in the current year
Land transfer income	X_7_	Proceeds from the sale of land by local governments
Added value of the secondary industry	X_8_	Final output of the secondary industry
Added value of the tertiary industry	X_9_	Final output of the tertiary industry
natural resources	X_10_	Number of employees in the mining industry

**Table 4:** Single-factor detection results of CLE in RBCs.

Variable	2005	2010	2015	2018
X_1_	0.28 (0.00)	0.27 (0.00)	0.32 (0.00)	0.40 (0.00)
X_2_	0.04 (0.41)	0.02 (0.65)	0.09 (0.03)	0.05 (0.27)
X_3_	0.40 (0.00)	0.47 (0.00)	0.48 (0.00)	0.49 (0.00)
X_4_	0.43 (0.00)	0.36 (0.00)	0.35 (0.00)	0.27 (0.00)
X_5_	0.43 (0.00)	0.31 (0.00)	0.45 (0.00)	0.35 (0.00)
X_6_	0.16 (0.06)	0.18 (0.00)	0.20 (0.00)	0.27 (0.00)
X_7_	0.19 (0.08)	0.34 (0.00)	0.20 (0.01)	0.19 (0.01)
X_8_	0.37 (0.00)	0.33 (0.00)	0.42 (0.00)	0.45 (0.00)
X_9_	0.40 (0.00)	0.51 (0.00)	0.48 (0.00)	0.06 (0.23)
X_10_	0.13 (0.02)	0.13 (0.02)	0.09 (0.08)	0.07 (0.39)

**Table 5:** Population growth rate and urbanization rate (%).

Area	Population Growth Rate				Urbanization Rate			
	2000–2005	2005–2010	2010–2015	2015–2018	2005	2010	2015	2018
All cities	3.58	3.56	3.44	2.07	34.04	42.60	54.04	57.75
NRBCs	5.05	5.09	4.96	2.96	34.53	43.41	55.21	58.67
RBCs	2.10	2.02	1.91	1.18	33.30	41.36	52.29	56.36

Note: Population growth rate data originate from World POP (www.worldpop.org (accessed on 12 October 2021)); urbanization rate data originate from the *China City Statistical Yearbook and Statistical Yearbook of Provinces and Cities*.

**Table 6:** Expansion rate of the different CL types in RBCs (%).

Land Type	1995–2000	2000–2005	2005–2010	2010–2015	2015–2018
Urban land	0.011	0.031	0.034	0.046	0.140
Rural settlement	0.005	0.003	0.001	0.011	0.022
Other construction land	0.017	0.066	0.065	0.182	0.102

**Table 7:** Factor interaction detection results of CLE in RBCs.

Factor Interaction	2005	2010	2015	2018
X_1_ ∩ X_2_	0.43 ↑	0.45 ↑	0.59 (*)↑	0.67 ↑
X_1_ ∩ X_3_	0.65 (*)↑↑	0.69 (*)↑↑	0.56 (*)↑↑	0.64 (*)↑↑
X_1_ ∩ X_4_	0.73 (*)↑	0.69 (*)↑	0.68 (*)↑	0.65 (*)↑↑
X_1_ ∩ X_5_	0.62 (*)↑↑	0.50 (*)↑↑	0.50 (*)↑↑	0.51 (*)↑↑
X_1_ ∩ X_6_	0.49 ↑	0.53 (*)↑↑	0.46 (*)↑↑	0.60 (*)↑↑
X_1_ ∩ X_7_	0.42 ↑↑	0.56 (*)↑	0.45 (*)↑↑	0.50 (*)↑↑
X_1_ ∩ X_8_	0.62 (*)↑↑	0.54 (*)↑	0.56 (*)↑↑	0.61 (*)↑↑
X_1_ ∩ X_9_	0.57 (*)↑↑	0.66 (*)↑	0.60 (*)↑↑	0.47 ↑↑
X_1_ ∩ X_10_	0.53 (*)↑	0.56 (*)↑	0.54 ↑	0.59 ↑
X_2_ ∩ X_3_	0.55 ↑	0.65 ↑	0.54 ↑↑	0.60 ↑
X_2_ ∩ X_4_	0.54 ↑	0.48 ↑	0.44 ↑↑	0.48 ↑
X_2_ ∩ X_5_	0.53 ↑	0.50 ↑	0.65 ↑	0.62 ↑
X_2_ ∩ X_6_	0.43 ↑	0.39 ↑	0.37 ↑	0.40 ↑
X_2_ ∩ X_7_	0.37 ↑	0.46 ↑↑	0.44 ↑	0.28 ↑
X_2_ ∩ X_8_	0.45 ↑	0.41 ↑↑	0.45 ↑↑	0.54 ↑
X_2_ ∩ X_9_	0.52 ↑	0.56 ↑	0.53 ↑↑	0.16 ↑
X_2_ ∩ X_10_	0.34 ↑	0.31 ↑	0.30 ↑	0.17 ↑
X_3_ ∩ X_4_	0.50 (*)↑↑	0.52 (*)↑↑	0.57 (*)↑↑	0.62 (*)↑↑
X_3_ ∩ X_5_	0.50 (*)↑↑	0.62 (*)↑↑	0.55 (*)↑↑	0.61 (*)↑↑
X_3_ ∩ X_6_	0.53 ↑↑	0.66 (*)↑↑	0.58 (*)↑↑	0.63 (*)↑↑
X_3_ ∩ X_7_	0.46 ↑↑	0.64 (*)↑↑	0.58 (*)↑↑	0.64 (*)↑↑
X_3_ ∩ X_8_	0.54 (*)↑↑	0.59 (*)↑	0.59 (*)↑↑	0.54 (*)↑↑
X_3_ ∩ X_9_	0.47 (*)↑↑	0.63 (*)↑↑	0.51 (*)↑↑	0.53 ↑↑
X_3_ ∩ X_10_	0.52 (*)↑↑	0.65 (*)↑	0.56 ↑↑	0.60 ↑
X_4_ ∩ X_5_	0.48 (*)↑↑	0.49 (*)↑↑	0.63 (*)↑↑	0.62 (*)↑↑
X_4_ ∩ X_6_	0.53 ↑↑	0.62 (*)↑↑	0.56 (*)↑↑	0.69 (*)↑
X_4_ ∩ X_7_	0.51 ↑↑	0.61 (*)↑	0.58 (*)↑	0.51 (*)↑
X_4_ ∩X_8_	0.49 (*)↑↑	0.48 (*)↑↑	0.55 (*)↑↑	0.56 (*)↑↑
X_4_ ∩X_9_	0.51 (*)↑↑	0.61 (*)↑↑	0.56 (*)↑↑	0.34 ↑
X_4_ ∩X_10_	0.55 (*)↑↑	0.62 (*)↑	0.58 ↑	0.39 ↑
X_5_ ∩ X_6_	0.53 ↑↑	0.58 (*)↑↑	0.55 (*)↑↑	0.51 (*)↑↑
X_5_ ∩ X_7_	0.51 ↑↑	0.55 (*)↑↑	0.54 (*)↑↑	0.51 (*)↑↑
X_5_ ∩ X_8_	0.56 (*)↑↑	0.53 (*)↑↑	0.54 (*)↑↑	0.57 (*)↑↑
X_5_ ∩ X_9_	0.52 (*)↑↑	0.60 (*)↑↑	0.56 (*)↑↑	0.45 ↑
X_5_ ∩ X_10_	0.56 (*)↑↑	0.52 (*)↑	0.62 ↑	0.49 ↑
X_6_ ∩ X_7_	0.37 ↑	0.48 (*)↑↑	0.42 (*)↑	0.50 (*)↑
X_6_ ∩ X_8_	0.50 ↑↑	0.45 (*)↑↑	0.52 (*)↑↑	0.56 (*)↑↑
X_6_ ∩ X_9_	0.49 ↑↑	0.59 (*)↑↑	0.56 (*)↑↑	0.34 ↑
X_6_ ∩ X_10_	0.46 ↑	0.44↑ (*)	0.44 ↑	0.45 ↑
X_7_ ∩ X_8_	0.51 ↑↑	0.55 (*)↑↑	0.53 (*)↑↑	0.55 (*)↑↑
X_7_ ∩ X_9_	0.48 ↑↑	0.57 (*)↑↑	0.57 (*)↑↑	0.30 ↑
X_7_ ∩X_10_	0.37 ↑	0.58 (*)↑	0.45↑	0.37 ↑
X_8_ ∩ X_9_	0.51 (*)↑↑	0.55 (*)↑↑	0.50 (*)↑↑	0.47 ↑↑
X_8_ ∩ X_10_	0.53 (*)↑	0.50 (*)↑	0.47 ↑↑	0.52 ↑↑
X_9_ ∩ X_10_	0.55 (*)↑	0.63 (*)↑↑	0.57 ↑↑	0.19 ↑

Note: * indicates passing the significance test, ↑↑ indicates mutual enhancement, and ↑ indicates nonlinear enhancement.

## Data Availability

For LUCCs data, please check https://www.resdc.cn/ (accessed on 18 October 2021); national, provincial, and municipal boundary vector data, not applicable; and for socioeconomic data involved in the indicators, please check https://data.cnki.net/ (accessed on 10 December 2021).
